# Effects of Trophy Hunting Leftovers on the Ranging Behaviour of Large Carnivores: A Case Study on Spotted Hyenas

**DOI:** 10.1371/journal.pone.0121471

**Published:** 2015-03-20

**Authors:** Gabriele Cozzi, Luca Börger, Pascale Hutter, Daniela Abegg, Céline Beran, J. Weldon McNutt, Arpat Ozgul

**Affiliations:** 1 Institute of Evolutionary Biology and Environmental Studies, Population Ecology Research Group, Zurich University, Zürich, Switzerland; 2 Botswana Predator Conservation Trust, Maun, Botswana; 3 Department of Biosciences, College of Sciences, Swansea University, Swansea, United Kingdom; Michigan Technological University, UNITED STATES

## Abstract

Human-related food resources such as garbage dumps and feeding sites have been shown to significantly influence space use, breeding success and population dynamics in a variety of animal species. In contrast, relatively little is known on the effects of unpredictable sources of food, such as carcasses discarded by hunters, on carnivore species. We evaluated the effect of elephant carcasses, mainly deriving from trophy hunting, on the ranging and feeding behavior of spotted hyenas (*Crocuta crocuta*) in the Okavango Delta, Botswana. Using data from hyenas monitored before and during carcass availability via GPS radio-collars and camera traps, we investigated changes in ranging and feeding behavior over time. Carcass availability influenced hyenas’ ranging behavior for an average of 10–12 days, after which their movements returned to patterns observed before carcass availability. In particular, we observed an increased spatial clustering of locations and reduced speeds (up to 15% less) between successive locations with carcass availability. Consistent feeding at carcasses during the first two weeks was typical, and some individuals fed from elephant carcasses for as long as 50 days. The impact and conservation value of hunting are often assessed based solely on the effects on the hunted species. Our results show that hunting remains can influence other species and suggest that such extra food could have important effects on critical life history processes and ultimately population dynamics. We recommend conservationists and wildlife managers evaluate management strategies and hunting practices regarding carcass disposal in order to incorporate the potential collateral impacts of hunting on non-hunted species in the same community.

## Introduction

Food availability is a major factor influencing many natural processes such as abundance, distribution, sociality and territoriality in wildlife species [1–5]. Recently, studies have increasingly investigated the effects of spatially and temporally predictable human-related sources of food such as garbage dumps, camping grounds, artificial supplementary feeding sites, urban areas, crop fields and livestock pastures on a variety of terrestrial and avian animal species [6–13]. These anthropogenic and predictable surpluses of food have been shown to significantly influence population densities and dynamics [14,15], reproductive success [16], behavioral adaptations [17], movement [18,19] and space use [20,21]. Few studies, however, have investigated the effects that large but spatially and temporally unpredictable sources of food, such as large carcasses deriving from trophy hunting, can have on carnivore species [22,23].

Biomass discarded by hunters might indeed represent an important food source for carnivore species. For instance, Ruth et al. [24] estimated that hunters discard approximately 500 tons of biomass each year in the Greater Yellowstone Ecosystem and hypothesized that these remains may provide an important source of food for bears prior to hibernation. Hence, whilst the conservation and management value of hunting has often been gauged on the direct effects it has on the hunted species and its socio-economic outcomes [25–28], there is a need for a more thorough understanding of how the hunting of one species can influence trophic dynamics in the broader community

Hunting of elephant (*Loxodonta africana*) (legal and illegal) has become an integral component of a complex range of management, conservation and economic policies in several African countries. Although hunting of elephants can impact entire ecosystems and regional elephant populations, management practices and hunting policies vary widely across elephant range states. Culling schemes have been implemented in some countries to manage elephant populations and prevent ecosystem degradation [29], while poaching activities have caused dramatic population declines elsewhere [30,31], and in still other countries trophy hunting represents an important management practice and a significant source of economic income and employment. All such activities produce a significant amount of discarded biomass in terms of elephant carcasses that, when added to those that die of natural causes, can have direct and indirect collateral effects on other single species and entire ecosystems. Given the size of an adult elephant, which can weigh between four and six tons [32], we hypothesize that the availability of elephant carcasses is likely to constitute a surplus of resources that can influence spatial ecology, feeding behavior, and population demography of carnivore species such as the spotted hyena (*Crocuta crocuta*).

Due to its unique ecological role, the spotted hyena has been given very high conservation priority [33]. Spotted hyenas are effective hunters and opportunistic foragers and have been reported feeding on a variety of items, including garbage in close spatial association with human populations [34–36]. They are one of the most specialized mammalian scavengers across the African continent, and are able to entirely consume elephant carcasses [37]. Even long bones are typically crushed and digested, providing access to the highly nutritious bone marrow [37,38] while only the skullcap, pelvis and parts of the spine remain unconsumed (G. Cozzi, pers. obs.).

In this study we investigated how an unpredictable large quantity of food, mainly deriving from elephant trophy hunting, altered the spatial and feeding behavior of spotted hyenas. We focused on changes in the spatial distribution and movement patterns of individuals in response to the sudden and unpredictable abundance of food from elephant carcasses. We considered elephant carcasses akin to “natural” food augmentation experiments for hyenas, as carcasses were unpredictably available in space and time. We expected hyenas to spend measurably increased time in the immediate vicinity of a carcass, leading to increased spatial clustering and reduced movement parameters (e.g. speed). We further calculated the number of extra days/year during which hyenas in the study area were able to feed from elephant carcasses. The analyses were performed on movement data collected by GPS radio-collars fitted on 12 spotted hyenas and on photographic data generated from camera traps located at elephant carcasses. These datasets allowed us to infer animals’ behavior over time both in the immediate vicinity of a carcass as well as elsewhere. We showed that leftovers from human hunting activities substantially influenced hyena ranging and feeding behavior for periods of several days to several weeks.

## Methods

### Study site and population

**Fig 1:**
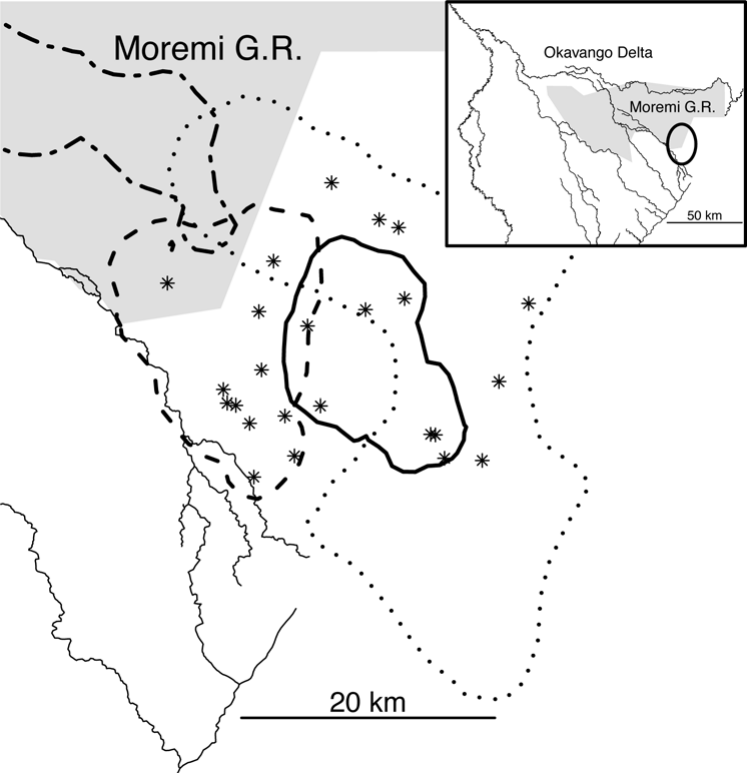
The study area (circle in the top-right inset) in the Moremi Game Reserve and the surrounding Wildlife Management Area, Okavango Delta, northern Botswana. Twenty-four elephant carcasses (asterisks) were visited by GPS radio-collared spotted hyenas. The carcasses were found within the territory (95% kernel utilization distribution) of three resident clans (solid, dash, and dot lines). Thin solid lines represent major rivers. The twodot-dash line in the top left corner represents part of the territory of a peripheral clan.

This study was conducted between 2008 and 2010 in the Okavango Delta in Northern Botswana, over a core study area of approximately 1’000 km^2^ (Fig. 1) as part of an ongoing, long-term project on African large carnivores. The area comprised the very southeastern section of Moremi Game Reserve (MGR), an unfenced protected area of 4’871 km^2^, and the adjacent governmental Wildlife Management Areas (WMAs), where the only permitted human activities are wildlife based tourism including trophy hunting—mainly of elephants. The region is characterized by a dry season between April and October and a wet season between November and March with an average precipitation of 450–600 mm/year [39]. The study area is also characterized by an annual flood, which typically peaks in June and subsides rapidly reaching the lowest level early in the year. The flood is out of phase with the wet season ensuring a continuous supply of water; as a consequence the majority of the herbivore species are relatively sedentary [40,41].

The local hyena population has been studied since 2006 and the density has been estimated at 14–15 individuals/100 km^2^ [42]. Yearly clan territory size is estimated at about 250 km^2^ and evidence suggests that clans number about 30 individuals plus dependent cubs at the den (G. Cozzi, unpubl. data).

### GPS data collection

A total of 12 hyenas were collared during the duration of this study, as part of an ongoing larger carnivore research project. Hyenas were immobilized and fitted with GPS radio collars (*GPS Plus*; Vectronic Aerospace GmbH, Berlin, Germany), which were scheduled to record eight GPS locations/day; one every two hours between 18:00 and 06:00 and one at 12:00. However, the collars of two hyenas consistently failed to record the noon locations. Collars were deployed for a mean duration of 362 days (min. 51, max. 595 days) with 47.2% of the locations collected during the hunting season (April–September). Overall, GPS acquisition success rate was > 90%.

Freely roaming hyenas where located and subsequently anesthetized via a dart filled with sedative fired from a CO_2_-powered dart rifle (*JM Special*, Dan-Inject ApS, Denmark). Drug composition and quantity used to anesthetize the animals followed approved and standardized protocols [43] and typically included a mixture of 50 mg of a tiletamin/zolazepam mix (*Zoletil*, Virbac, South Africa), 3 mg of medetomidine (*Domitor*, Kyron Laboratories, South Africa) and 50 mg of ketamine (Kyron Laboratories, South Africa). This procedure did not require any form of capturing or trapping of the animals. During anesthesia we recorded the general health of each sedated animal, took body measurements and collected blood samples. All sedated individuals safely recovered from the anesthesia and showed no injuries or signs of distress, for further details see [44]. Immobilizations were carried out with the assistance of a Botswana registered veterinarian and in accordance with Botswana laws. All procedures were approved and conducted under research permit EWT 8/36/4 granted to G.C. by the Botswana Ministry of Environment, Wildlife and Tourism.

### Elephant carcasses

Elephants are very abundant in Botswana, where the population is estimated to exceed 100’000 individuals [45]. Following the Botswana law, elephant trophy hunting was allowed each year between April and September, before being completely banned at the end of 2013. During the study period, a maximum quota of 14–17 individuals/year was allowed within the governmentally designated hunting areas that overlapped with our core study area. The quota was assigned by the Botswana Government to professional hunting companies operating in the area and independently of this study. The professional hunters informed us of the dates and locations of the elephants that they shot; no animals were killed specifically for this study. The tusks were always taken as main trophy, and in few cases the feet, the ears or the trunk were removed. Other carcasses of elephants that died of natural causes were discovered by tracking radio-collared hyenas. Furthermore, during the entire duration of the study, clusters of GPS locations collected from radio collar data were regularly investigated and such clusters invariably corresponded either to den sites or elephant carcasses. This suggests that few, if any, carcasses went undetected.

The majority of the carcasses (24 out of 29) laid within the territory of known and regularly monitored clans of spotted hyenas (Fig. 1). Territories were calculated as 95% kernel utilization distribution (UD), a robust density estimation procedure [46], using with the ‘kernelUD’ function in the ‘adehabitatHR’ package in R (The R Foundation for Statistical Computing; version 3.0.3). The reference bandwidth (*h*_*ref*_) was chosen as initial smoothing parameter. Then, since with clumped data *h*_*ref*_ is likely to over-smooth the data [47,48], the final smoothing parameter *h*_*final*_ was chosen on the basis of visual assessment of each kernel UD produced with alternative *h*-values [49–51], in order to exclude biologically unrealistic areas such as inaccessible landscape (i.e. rivers and swamps). Note also that *h* computed by Least Squares Cross Validation typically fails or under-estimates the UD for GPS data, especially when animal locations data are very close together and hence is not recommended anymore for tracking data [51,52]. This procedure enabled us to define, for each hyena, whether a carcass was within or outside its territory (note that for hyenas territories are equivalent to defended home-ranges and hence density estimates using location data are adequate [51,53]). The remaining five carcasses were found outside the territorial boundaries of the known hyena clans, and were not visited by the GPS radio collared individuals; these carcasses were therefore not considered for further analyses.

### Camera trapping

**Fig 2:**
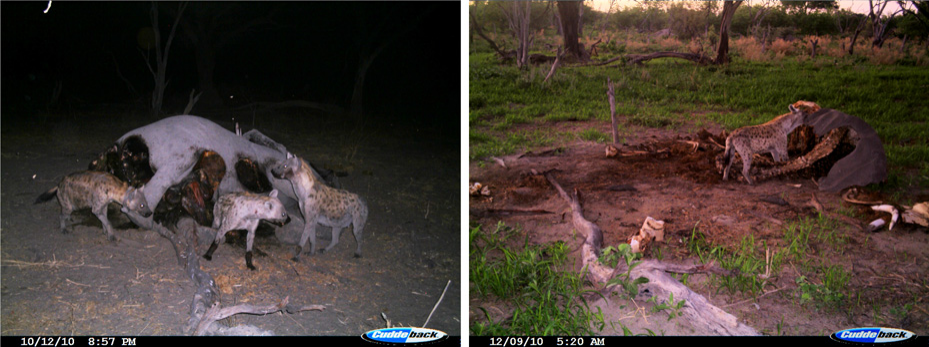
Camera trap pictures taken two (left) and 59 (right) days after elephant death. After two days the soft tissues around the belly have been accessed and after 59 days only the spine, the tusks and pieces of dry skin are left behind (note the lying branch for spatial reference). Dates are given in mm/dd/yy.

Camera traps (*Cuddeback Expert*; Cuddeback, Wisconsin, USA) at elephant carcasses were attached to trees or shrubs at about 1.5–2 m above ground to be out of reach of the hyenas and about 15 m from a carcass to ensure an adequate view of field. Given the nocturnal activity patterns of hyenas [54] and to avoid day-time battery use, cameras were set to take pictures between sunset and sunrise only. Date and time were recorded for each picture (Fig. 2). Five carcasses were monitored with camera traps for a total of 120 carcass-nights.

Because we wanted to understand changes in the number of individual hyenas visiting the carcasses and in the time they spent feeding at the carcass, individual hyenas were identified based on their unique coat patterns. This was done for all pictures (n = 2,965) taken at two carcasses only; where a total of 23 and 25 individuals could be identified. At the other three carcasses, the number of hyenas present on each picture was noted, but individual hyenas were not separately identified. Analysis of the two carcasses where individuals were identified yielded a full count of the number of hyenas visiting a carcass each night and the time they spent at the carcass. This was defined as the time difference between the first and the last picture taken for any given individual each night. We found a strong positive linear relationship (adjusted R^2^ = 0.85) between the number of individually recognized hyenas present each night and the number of hyenas on the most populated picture (i.e. the picture with the largest number of individuals) on the same night (S1 Fig.). We therefore subsequently used the estimated coefficient for slope (b = 1.97, p < 0.001) in the following equation:

$${\text{Y}}_{\text{n}}=\text{ 1}.\text{97 }*{\text{ X}}_{\text{n}}$$

to infer the number of single individuals during night n (Y_n_), given the number of hyenas in the most populated picture during the same night (X_n_), for the remaining three carcasses. The intercept was not significantly different from 0 and was therefore not included.

### Statistical analysis

We investigated the hyenas’ *behavior at elephant carcasses* by integrating GPS movement data from radio collars and photographic data from camera traps placed at the carcasses. The influence of elephant carcasses on the hyenas’ *ranging behavior* was analyzed by means of GPS movement data only. The R Software Environment for Statistical Computing (version 3.0.3) was used for all analyses.

1 **Behavior at the carcass**. To investigate the likelihood of hyenas visiting an elephant carcass—with potential consequences on their movement and feeding behavior—we fitted a generalized linear mixed model (with a binomial distribution) with *presence/absence* as response variable. Presence was given a value of 1 if, within a day, at least one GPS location was within 500 m from a carcass. This distance was set under the assumption that hyenas are well aware of an elephant carcass at such distance. This radius also allowed detection of individuals that only briefly visited the carcass and subsequently withdrew to feed on small scraps or to rest. The *number of days after a carcass was available* was treated as the main explanatory variable. The other covariates included were *denning* (i.e. whether a hyena had depended cubs at the den or not), *hunting period* (i.e. the hunting season was divided in three equally-long periods of two months: early, middle and late season), *territory* (whether a carcass was within or outside the territory of a given hyena) and *burned* (i.e. whether a carcass was burned or not). Some carcasses were burned few days after the killing because the carcasses were in sight of tourist roads or near surface water table (the burning was then necessary to avoid bacterial contamination). Carcass and hyena identities were treated as random terms. The model was implemented using the ‘glmer’ function in the package ‘lme4’ in R (The R Foundation for Statistical Computing; version 3.0.3). On some occasions the local community would collect part of the flesh for subsistence purposes; it was, however, not possible to quantify the amount of flesh removed and this covariate was therefore not included in the analyses. The majority of the elephant was, however, left untouched.

We used data from camera traps to investigate the influence of *days after a carcass was available* and *hunting period* on the Poisson-distributed response variable *number of individual hyenas present* at a carcass. Elephant identity was treated as random term and a Poisson distribution was used in a GLMM framework (‘lme4’ package in R). Camera traps were removed when a carcass was burned, so we were not able to test the effect of this practice (*burned*) on the number of hyenas present. Whether hyenas photographed at the carcass had den-dwelling cubs (*denning*) and whether they were members of the local clan (*territory*) could not be assessed and these two variables were consequently not used.

We modeled the *time spent at a carcass* as a function of time using a generalized additive mixed model, which allows for considerable flexibility where a non-linear relationship between the response and predictor variable is expected [32,55]. Carcass and hyena identities were treated as random terms. For this analysis only data from the two carcasses where individual hyenas were identified based on their unique coat patterns were used. Because the maximum possible length of stay each night corresponded to the time difference between sunset and sunrise (i.e. the very fist and the very last possible picture), we treated the response variable as proportion data by dividing the length of stay by the maximum possible length of stay. We accordingly used a binomial distribution [55].

1 **Ranging behavior**. We used GPS movement data to investigate the influence of elephant carcasses on the hyenas’ spatial distribution and movement patterns. Methods such as kernel utilization distribution maps have been traditionally used to investigate concentration-dispersion dynamics and animal spatial distribution associated with clustered food resources [20,56]. However, given the limited number of available locations (maximum of eight locations/individual/day), we developed an alternative statistic: For each individual and for each day, the average pairwise distance among all recorded locations was calculated. This daily value was taken as a measure of the degree of scattering/clustering and used to investigate the hyenas’ spatial distribution. In addition, the average speed between consecutive locations was used to investigate movement behavior before and after an elephant carcass was deposited. This movement statistic was preferred to step-length (i.e. the distance between consecutive locations) because it is less sensitive to missing values (since the time between consecutive locations is taken into consideration). We restricted our analyses to a period of 20 days before and after a carcass was deposited to isolate the effect of the carcass from other confounding ecological, social, environmental and individual conditions, which are likely to change over a longer period of time. A generalized additive mixed model framework was used to capture changes in *scattering* and *speed between locations* over time before and after a carcass was deposited. We expected both response variables to change in response to a perturbation, i.e. the sudden availability of a carcass, and to slowly return to original levels after some time. Both *scattering* and *speed* were analyzed as a function of a smoother of *time* and hyena identity was treated as random term. *Scattering* parameter and speed were square root transformed to meet normality of residuals. The model was implemented in R using the ‘gamm’ function in the package ‘mgcv’. For all statistical analyses, model simplification followed a backward selection procedure based on the Akaike Information Criterion [55].

## Results

### Elephant carcasses availability

**Fig 3:**
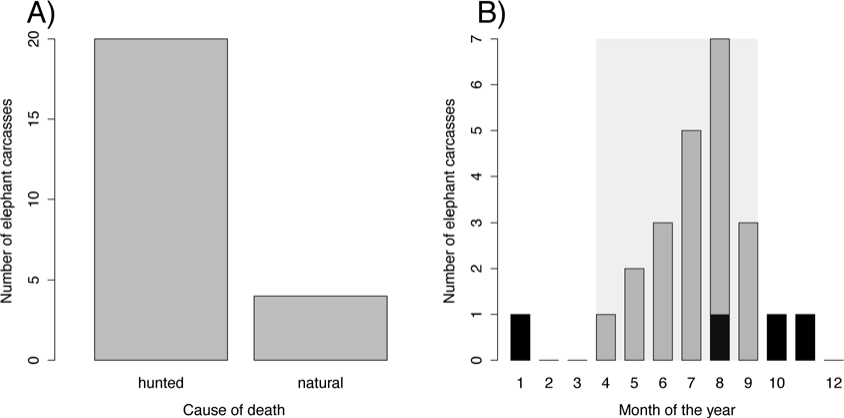
Availability of elephant carcasses between 2008 and 2010. In B) the grey bars represent trophy hunted elephants, black bars represent naturally dead elephants and the pale-grey polygon shows the hunting season.

Despite the large number of free ranging elephants in the study area, the number of carcasses available to spotted hyenas was highly dependent on the number of elephants shot for trophy hunting. Only 5 of the 29 detected carcasses (17.2%), and 4 of the 24 carcasses (16.7%) considered for the analyses (see Methods) were elephants that died of natural causes (Fig. 3). Under the assumption that natural elephant mortality is evenly distributed throughout the year, during the hunting season (April–September) an average excess of about seven elephant carcasses was available yearly in the study area (Fig. 3b). This is roughly equal to an additional biomass of 28–42 tons.

### Behavior at the carcass

**Fig 4:**
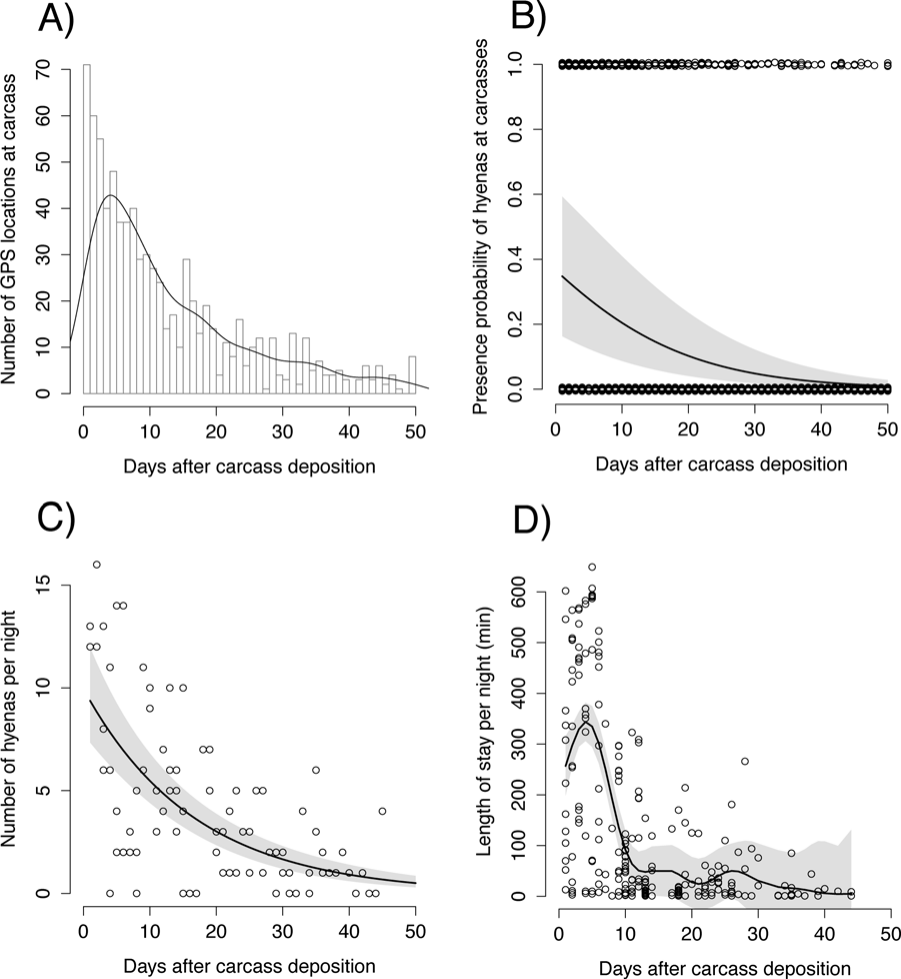
Use of elephant carcasses by spotted hyenas as a function of time after carcass deposition inferred by means of GPS data (A and B) and camera trapping (C and D). Histogram of the number of GPS locations from all radio-collared hyenas (A) and likelihood of finding hyenas (B) within a radius of 500 m from elephant carcasses (n = 24). In A, the line represents the density estimates. In C, data from all five carcasses monitored with camera traps were used. In D, data from the two carcasses where each single individual hyena present had been identified were used (see text form more details). Grey shaded areas represent confidence intervals. Dots represent raw data. Jittering has been introduced in B for easier visualization.

Over the three years, seven of the 12 collared hyenas visited one or more carcasses. We detected a high consistency between the results obtained from GPS data (Fig. 4a-b) and camera trap data (Fig. 4c-d). As expected, the likelihood of finding hyenas at a carcass significantly decreased over time and after 12 days the initial value was reduced by 50% and was close to nil after about 50 days (Fig. 4a-b). Carcasses within an individual’s territory were more likely to be visited (z = 3.95, p < 0.001), while burning of the carcasses had a direct negative effect on the hyenas’ presence probability (z = -2.46, p = 0.01). Whether a hyena had dependent cubs at the den or not had no significant effect on her presence at a carcass. We did not detect significant differences in the probability of presence among the early (April-May), middle (June-July) and late (August-September) periods of the hunting season, suggesting that carcasses are equally attractive throughout the season. The number of hyenas visiting a carcass each night also constantly decreased over 50 days (Fig. 4c). Time spent at carcass significantly decreased over time (F_edf = 7.1_ = 24.46, p < 0.001) and reached a plateau at 12 days, after which hyenas spent less than one hour/night at a carcass (Fig. 4d). A decrease in carcass quality and hence profitability over time resulted in shorter rather than longer visits; the latter scenario can be expected if the same energetic gain has to be extracted from a low-quality and difficult-to-process food source.

### Ranging behavior

**Fig 5:**
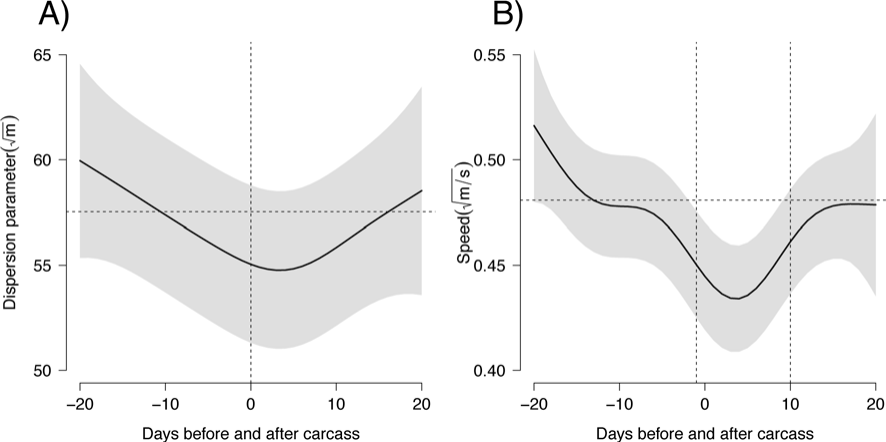
Scattering parameter (A) and movement metrics (B) immediately before and after a carcass was available. The horizontal dotted line represents the mean predicted value during the 20 days prior to when a carcass was available. The grey shaded areas represent confidence intervals. The vertical dotted lines indicate the time during which speed (12 days) is consistently below the average value recorded for the 20-days period prior to carcass availability.

We did not detect any differences in ranging behavior between the hunting and non-hunting season, suggesting that few carcasses in excess are unlikely to significantly alter the hyenas’ spatial behavior over a large time scale of 6-months. We could instead detect a significant difference in scattering parameter (F_edf = 2.4_ = 3.90, p = 0.016) and speed (F_edf = 4.4_ = 4.80, p < 0.001) over a shorter time scale. In particular, speed was consistently below average for 12 days when carcasses were available (Fig. 5). This window of time likely represent the actual temporal span over which carcasses consistently influence hyenas’ ranging patterns. During this period, we observed a 15% reduction in mean speed compared to that before carcass deposition.

## Discussion

We investigated the effects of elephant carcasses mainly derived from trophy hunting on a large carnivore species, the spotted hyena. We showed that elephant carcasses substantially influenced the ranging and feeding behavior of focal hyenas for at least 10–12 days (Fig. 4d and 5). After this time carcass value probably decreased and hyenas ranging behavior returned to patterns observed prior to carcass availability (Fig. 5). However, the period of carcass utilization varied widely and some individuals continued to feed on elephant carcasses for as long as 50 days (Figs. 2b and 4). Considering that the great majority (83.3%) of the carcasses were hunted elephants, our results show how the remains of human hunting activities can significantly affect the behavior and ecology of a carnivore species.

The observed sharp decrease in time spent at the carcass after 10–12 days might correspond to decreasing accessibility of meat due to deterioration and consumption. As carcass quality deteriorated over time, shorter visits were observed, rather than longer visits, which would be required to access an equal amount of energy from comparatively difficult-to-process resources such as bones and skin, This finding, and the observed lack of differences between early, middle, and late hunting season, suggest that hyenas in the highly productive Okavango Delta can find alternative sources of food relatively easily. Alternatively, since the remaining difficult-to-process resources are both inaccessible to species other than hyenas and less likely to deteriorate further or disappear, we could expect less urgency in accessing them immediately. Given the observed effect of carcasses on hyenas foraging and movements in the comparatively rich Delta habitats, we expect that in drier habitats the carcasses of hunted large animals would represent an even more important food resource for carnivores, and we hypothesize a more pronounced and longer-lasting behavioral response. A detailed evaluation of the effect of unpredictable spatially and temporally clumped resources, including management of carcasses, may be particularly important and recommended in such environments. Regardless of the environment, large carcasses constitute a rendezvous point for social purposes and an important source of food for lower ranking individuals for a longer time [20,57,58].

Considering an average clan size of 30 adults for the study area, a mean daily requirement of 5 kg of biomass/hyena/day [59,60] and a feeding time of 12 days/carcass, an estimated total of 1800 kg of biomass are consumed from an average elephant carcass. In view of unknowns and approximations (e.g. actual mass of the elephant carcass, weight of the plant material in the digestive tract, discarded and non-digestible parts, biomass removed by other saprophagous species such as vultures, and meat harvested by the local villagers), this estimate seems realistic for an adult elephant with a mass > 4 tons, thus supporting our conclusion that carcasses significantly influence hyena feeding and ranging behavior for periods of at least 10–12 days. On average, the three hyena clans in this study each profited from three carcasses per year, which translates into a total of 30–36 extra feeding days/year/clan derived from trophy hunting. Under the assumption that daily energetic requirements are relatively constant throughout the year, this period of time is equivalent to a substantial 8.1%–10.0% of the annual budgetary intake. This surplus of food can be expected to have positive effects at the individual and population levels. Following the ban on elephant hunting imposed in 2013, clan size may decrease in the future as a result of reduced food availability. Such reduced food availability could result in increased hyena hunting pressure on natural prey species and increased indirect competition with other carnivores. Despite speculative nature, these scenarios illustrate how hunting leftovers could have collateral effects on other species in the community.

Detailed information on the social dominance structure of the clans in this study that would enable us to determine whether dominance affected feeding and access to carcasses was unavailable [20,61]. Monopoly of food resources by dominant group members [61] could have been responsible for the prolonged (> 50 days) use of carcasses by some (subordinate) members. It is, however, not clear whether dominance would be expected to affect feeding behavior at such ‘super abundant’ food resources or whether “participants eat peacefully together” as reported by Kruuk [37]. Additional observations suggest that such a substantial source of food can nonetheless have important impacts on between-groups interactions. For instance, at least 10 carcasses were visited by members of two clans and one by members of three clans, though not simultaneously. On one occasion an old female traversed the territories of two clans covering 37 km in 10 hours (S2 Fig.) to reach a carcass where she fed next to members of the resident clan for eight consecutive days before going back to her territory. Despite the hyenas’ remarkable sense of smell, it is almost certainly impossible that she detected the carcass from such a distance. We speculate that this old individual learned to leave her clans’ territory from time to time to investigate the area where elephant hunting occurred until she eventually picked up the scent of a carcass from a closer distance. This hypothesis is strengthen by the fact that the same hyena visited elephant carcasses well outside her territory on three other occasions (S2 Fig.). These findings show how large carcasses can influence long-distance ranging patterns and extra-territorial forays in large carnivores. Similar extra-territorial foraging forays have been observed elsewhere but these were due to natural and predictable fluctuations in food resources [58,62], rather than to the presence of an anthropogenic and unpredictable abundant source of food.

Our models assumed that hyena visits during consecutive days were independent of one another. Negative correlation (i.e. due to satiety) could, however, result in more time allocated to alternative activities such as patrolling of the territory. This would cause an increase in scattering parameter and speed partially explaining the noise observed in the data and the relatively large confidence intervals (Fig. 5) intervals. Additional ecological, environmental, and social factors such as the presence of alternative sources of food, decomposition through microbial activity, intra and interspecific interactions at the carcass, individuals’ social ranks, and the amount of meat harvested by the local community were likely to contribute to the high variation in patterns of carcass utilization observed (Figs. 4 and 5). In particular, given the relatively large confidence interval, caution is required in the interpretation of the effect of elephant carcasses on scattering parameter (Fig. 5a) from the gamm model (see for example [55] for a more detailed discussion). Although this does not challenge the validity of the results presented here, we note the value of conducting similar studies with larger sample size.

Other carnivore species (e.g. side-striped jackal *Canis mesomelas*, lion *Panthera* leo, honey badger *Mellivora capensis*) have been observed feeding at the carcass. This suggests that the concepts applied to our specific system can be extended to other carnivore species [23]. The presence of carcasses thus acts as an attraction and contact point for multiple species and for individuals of neighboring groups. This could facilitate the spread of diseases between species and among groups, and hence have important ecological, conservation and management implications [63,64]. Investigation of how hunting activities and hunting leftovers can influence community assemblies and the ecology of diseases is therefore highly recommended and should become integrated into hunting policy and practice.

## Conclusions

While the conservation value of hunting is often gauged on the effects that it has on the hunted species, we identified a lack of research on the influence of hunting leftovers on non-hunted species. Our study is the first of its kind to investigate the direct consequences of spatially and temporally unpredictable large sources of food, mainly deriving from human hunting activities, on a large carnivore species. We demonstrated that remains from elephant trophy hunting substantially influence the spatial and feeding behaviour of spotted hyenas for up to several weeks and estimated that these leftovers constituted 8.1%-10% of the yearly individual feeding budget. Furthermore, we showed that acting as attraction point, hunting leftovers can influence intra and interspecific interactions and community assembly. Such a significant surplus of resources could have important effects on reproductive timing and frequency, adult and offspring survival and ultimately population dynamics of carnivore species. Similar effects on species demographic traits have been shown for predictable human-related sources of food such as garbage dumps [14,16]. Considering the large amount of biomass discarded each year by hunters [24], there is great potential for further investigations of these previously unacknowledged but significant impacts on other species in the community. This is particularly important for large carnivores, which have been shown to significantly influence ecosystem dynamics through the effects of trophic cascades and whose conservation status is often threatened [33]. Thus, an in-depth understanding of the relationship between hunting remains and carnivores is essential for an accurate evaluation of alternative management practices, such as removal or abandonment of carcasses. The addition of this dynamic will provide a more informed basis for further discussion and development of management policies related to trophy hunting and the potential impacts on sympatric carnivores.

## Supporting Information

S1 Fig Relationship between the number of individually recognized hyenas present each night and the number of hyenas on the most populated picture (i.e. the picture with the largest number of individuals) on the same night. This investigation was done for two carcasses only, and the obtained relationship used to infer the number of single hyenas for the other carcasses, where only the number of hyenas present on each picture was noted but individuals were not uniquely identified.
(TIF)

S2 Fig Example of one extra-territory foray by one female hyena that visited elephant carcasses outside her clans’ territory (dot-dash line). The open circles connected by solid line represent consecutive GPS locations of her 37 km and 10 hours long trip across the territory of two unrelated clans (two-dash, dash). This trip, with starting and ending times given in the figure, ended at an elephant carcass (asterisk) where she was observed feeding next to members of the local clan. The same hyena visited carcasses outside her territory in three other occasions. The four shared carcasses are indicated by arrows. Only carcasses (asterisks) available during the period in which this female was monitored are shown.
(TIF)
