# Supplementary figures and images for: Effects of Trophy Hunting Leftovers on the Ranging Behaviour of Large Carnivores: A Case Study on Spotted Hyenas
Source: PLoS One. 2015 Mar 20;10(3):e0121471. doi: 10.1371/journal.pone.0121471 (PMC4368814; doi:10.1371/journal.pone.0121471)

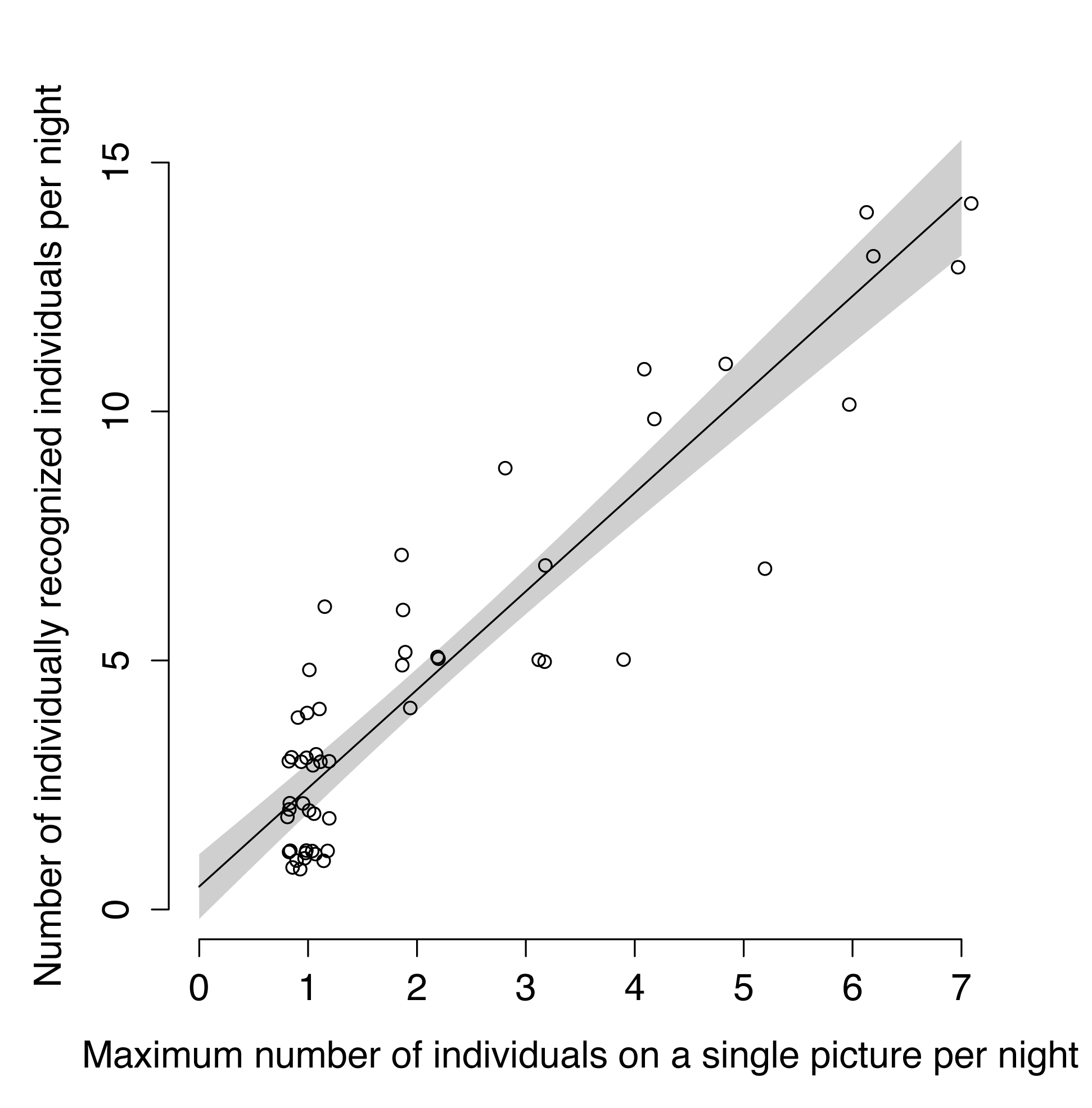

Supplement: S1 Fig — This investigation was done for two carcasses only, and the obtained relationship used to infer the number of single hyenas for the other carcasses, where only the number of hyenas present on each picture was noted but individuals were not uniquely identified. (TIF) [file pone.0121471.s001.tif]

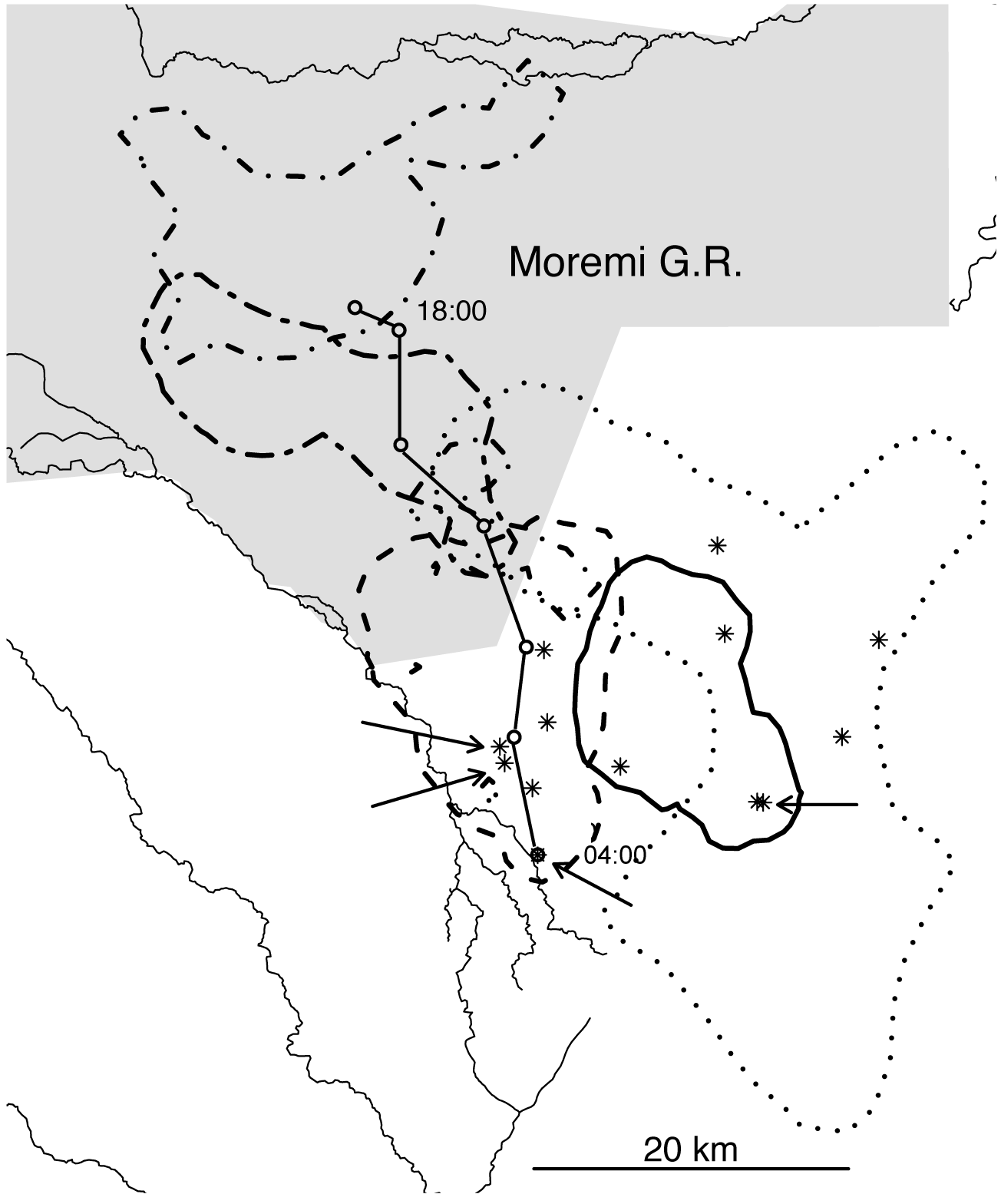

Supplement: S2 Fig — The open circles connected by solid line represent consecutive GPS locations of her 37 km and 10 hours long trip across the territory of two unrelated clans (two-dash, dash). This trip, with starting and ending times given in the figure, ended at an elephant carcass (asterisk) where she was observed feeding next to members of the local clan. The same hyena visited carcasses outside her territory in three other occasions. The four shared carcasses are indicated by arrows. Only carcasses (asterisks) available during the period in which this female was monitored are shown. (TIF) [file pone.0121471.s002.tif]
